# Examining the impact of green technological specialization and the integration of AI technologies on green innovation performance: evidence from China

**DOI:** 10.3389/frai.2023.1237285

**Published:** 2024-04-25

**Authors:** Sirinant Khunakornbodintr

**Affiliations:** Sino-Danish College, University of Chinese Academy of Sciences, Beijing, China

**Keywords:** green technologies, artificial intelligence, technological specialization, innovation performance, technological distance, relatedness, AI integration

## Abstract

China's commitment to achieving carbon neutrality by 2060 has sparked scholars' interest in examining the environmental ramifications of green technologies in the digital era. While plenty of them provide eco-efficiency policy such as increasing R&D investment or stimulating green exports, little attention has been paid to the firm-level technological management and recombination strategies such as differentiation/specialization of green portfolios along with AI integration, which can significantly impact the pace of net-zero transitions. To address these gaps, this study investigates the moderating effect of technological specialization on levels of AI integration into green technologies estimated by green-AI technological distance and enterprises' innovation performance in Chinese contemporary contexts. Regression results of fixed-effect model in Chinese patent data (2011–2020) indicate that enterprises' green innovation performance is significantly improved as AI integrates more into the green technologies due to the legitimacy and the inability to appropriate more green values. Interestingly, specialized green-technological enterprises demonstrate superior performance in integrating distant AI technologies. This occurrence could potentially be driven by the governments' incentives and the organization's risk attitudes, shaping green innovation outcomes. Hence, the study underscores the importance of considering both the AI integration and green specialization in shaping innovation outcomes amidst green transitions.

## 1 Introduction

Green initiatives have prompted the development of technologies leveraging digitalization to promote environmental sustainability by departing the innovation from existing technological portfolios (Montresor and Quatraro, 2020; Ahmad et al., 2021; Chen et al., 2022). However, the extent to which each green sector has been integrated with artificial intelligence (AI) technologies and their impacts on firm innovation performance is unknown. Firms are heterogeneous in their innovative capacity and resources, which lead to different strengths in integrating simple and complex AI technologies, respectively (Caviggioli et al., 2023; Li et al., 2023). The current literature lacks appropriate innovation strategies for heterogeneous firms to manage complex technological integration.

AI technologies can process big data and predict future scenarios based on existing information. It has the potential to solve climate problems and create paths toward sustainable development goals, enabling organizations to explore more distant, unrelated technologies (Montresor and Quatraro, 2020). While AI offers numerous benefits, its flexible boundaries result in heterogeneous outcomes across regions, technologies, and industries (Kopka and Grashof, 2022). As AI's features continue to evolve, they reshape market dynamics and increase in complexity (Nambisan, 2017; Dou and Gao, 2022). Some enterprises may struggle to adapt, assimilate, and integrate complex AI technologies. Given the recent techno-economic paradigm shift driven by climate change (Santoalha et al., 2021), the strategic hybridization of green and AI technologies in specialized green-technological enterprises (SGTEs) for innovative impact requires further exploration. In light of these research gaps, an important question is raised: Do SGTEs integrate related (proximate) AI technologies, and why?

In this study's context, organization capabilities are proxied by their technological specialization, which refers to the build-up of an organization's competitive advantage in specific research domains or sectors (Balland et al., 2019). By calculating the proportion of the share of a given industry in a given enterprise over the share of this industry in all enterprises in China, the index provides information about the degree to which the enterprise is devoted to a specific industry not presented in any other enterprises. Meanwhile, the integration of AI could be measured using technological distance which determines the commonalities in green and AI technologies, signifying the cross-section of technological knowledge of the inventors.

The relatedness theory suggests that firms integrating new AI technologies related to existing green knowledge are more likely to exhibit greater green innovation performance than those integrating AI technologies less related to existing green knowledge, due to the inherent risks and difficulties involved (Neffke et al., 2011; He et al., 2018; Balland et al., 2019). However, with active policy support and the involvement of a multitude of agents, green entrepreneurs may take risks in integrating unrelated technologies by leveraging special resources and capabilities to pursue disruptive innovations (Ning and Guo, 2022). Related activities have cultivated regional specializations, attracting highly capable firms to invest in the areas but technologically specialized enterprises may have varying approaches to integrating AI technologies in the green industry.

This research emphasizes the importance of conducting relevant studies within the context of Chinese enterprise for two primary reasons. Firstly, China's institutional environment is unique and has achieved notable success in implementing various green sectors, such as hydropower, biomass, and solar PV (Lema et al., 2020). However, the intricate relationship between AI development and green technologies presents a critical challenge in China, impacting its structural transition and innovation (Yin et al., 2023). Furthermore, the outcomes of ecological footprints caused by a variety of country-level factors vary across different levels of institutional quality (Rafei et al., 2022). Secondly, China seems to overcome the ‘environmental resource curse' where countries experience high carbon dioxide emissions and natural resource consumption at the early stage of economic development, the time when firms struggle to find a balance between financial pursuits and environmental legitimacy (Wang et al., 2015; Chen et al., 2022; Jahanger et al., 2022). Recently, many Chinese enterprises increasingly adopt environmental labeling certification which leads to higher corporate environmental innovation (Ren et al., 2022).

Running fixed-effects regression models based on green and AI patent data obtained from the Bureau van Dijk (BvD) Orbis Intellectual Property (IP) database spanning from 2011 to 2020, this study aligns with the prevailing relatedness theory that the integration of proximate AI technologies enhances green innovation performance measured by the number of green patents. Contrary to hypothetical arguments though, SGTEs exhibit superior performance when AI technologies are distant from green technologies. This finding challenges the assumption that SGTEs would perform better when AI technologies are closely related to green technologies, considering the inter-dependencies among similar agents and high-risk perception. Instead, SGTEs excel when AI technologies are more detached from green technologies. Two theoretical explanations are provided for this phenomenon. From a competition-based view, enterprises attempt to influence the resolution of uncertainty in their favor over the competitors by increasing their specialization (Toh and Kim, 2013). This is connected to the attribute of SGTE managers whose risk-averse attitude increases their awareness of integrating distant and complex AI technologies, thereby achieving higher innovation performance. The government's favor for SGTEs also provides them an advantage to take more risks (Zhang and Zhang, 2023). Lastly, the research reveals that Chinese enterprises tend to generate more AI technologies alongside the development of green technologies when these technologies are closely related. Consequently, this study sheds light on the competitive advantages and limitations of SGTEs when engaging with AI technologies.

This study makes three key contributions to existing literature. Firstly, it provides empirical evidence that aligns with the relatedness theory, confirming that enterprises achieve high innovation performance in green innovation when AI technologies are closely related to green technologies. Secondly, it contributes to the specialization theory by highlighting the significance of SGTEs in future development. These enterprises possess the ability to recognize the advantages offered by distant AI technologies. Lastly, this research offers policy and management recommendations for the evaluation of green technologies with substantial potential for AI integration, aligning with the Sustainable Development Goal (SDG) initiatives in the digital era.

The remainder of this study is organized as follows: Section 2 presents the theoretical background and hypotheses. Section 3 elaborates on the methodology, whereas the results are presented in Section 4. Section 5 discusses the implications, limitations, and future research prospects. Finally, Section 6 summarizes the discussions into conclusions.

## 2 Literature review and research hypotheses

### 2.1 The role of AI in China's green industries

In 2017, China unveiled its “Next Generation Artificial Intelligence Development Plan” aiming to position itself as a global AI innovation center by 2030 (CAST, 2017). With renewable energy production targets set in the 11th Five-Year Plan (FYP, 2006–2010) and specific provisions for green investments in the 12th Five-Year Plan (FYP, 2010–2015), AI has become increasingly crucial to drive green innovation in China. As the government continues to support AI and green technological development, sustainability has garnered attention from business managers across various sectors, including energy and ICT.

So far, AI is recognized for its general-purpose properties that facilitate the recombination of knowledge, enabling firms to bridge sectoral technological gaps (Qiu and Cantwell, 2018). General Purpose Technologies (GPTs) such as AI exhibit horizontal applications, transformative potential, pervasiveness, and complementarities that contribute to innovation (Bresnahan, 2010). GPTs widen the sets of knowledge items and enable individuals to master more complex technologies while vertically extending knowledge items through the co-occurrence of specific applications (Cicerone et al., 2023).

Despite the government's incentives and the appreciation of GPT advantages, businesses engaged in green innovation face the dilemma of whether to adopt AI technologies. There are risks and concerns regarding organizational secrets exposure, poor management, and excessive digital infrastructure (Dou and Gao, 2022). Additionally, Chinese AI scientists are specialized in segregated areas, resulting in technological gaps in less popular technologies (Barton et al., 2017). While some studies have examined the integration of AI technologies into green innovation in the form of 5G and the Internet of Things (Yin and Yu, 2022), the technological distance represented by organizations' ability to transfer AI knowledge to green technologies through inventions also plays a crucial role in green innovation.

Current research trends primarily focus on manufacturing firms' environmental responsibility and external factors, contributing to national dual-carbon policies but overlooking firms' future innovation plans. Although studies indirectly suggest that firms can gain green legitimacy through increased adoption of robotics and AI-related academic publications (Wang et al., 2015; Zhang and Wu, 2021; Liu et al., 2022a,b), the extent of AI applications in the green industry varies across sectors, developmental stages, and firms' capabilities (Kang and He, 2018). Research on internal factors and their impact on green-digital integration is limited, mainly within the manufacturing industry.

Digital and green technologies are interconnected through digital literacy, which refers to the workforce's competencies in researching, communicating, planning, and organizing AI technologies, including ICT infrastructure (Santoalha et al., 2021). The lack of such skills, resistance from staff, long implementation time, and technical complexity are barriers to reaching efficient operations (Ahmad et al., 2021). Consequently, firms' innovation capabilities in the green industry rely on their workforce's ability to recognize and develop AI applications within green technologies.

Overall, despite the significant support provided by the Chinese government to address pollution through emerging green technologies, the role of AI in the green industry remains relatively unexplored. There is a scarcity of research investigating the technological distance between two separate technological domains. Existing studies primarily examine the influence of exogenous factors on firms' environmental performance, overlooking endogenous factors and their impact on innovation performance.

### 2.2 The main effect of green AI technological distance on innovation performance

Technological distance, an antonym of technological relatedness, refers to two technologies sharing a high degree of commonality in knowledge bases that derive from mutual scientific principles or cognitively similar industries (Hidalgo et al., 2007). The stylized fact of technological distance in the relatedness theory is that innovating new products based on what's available in the basket (portfolio) would yield optimal advantages (Balland et al., 2019). Short technological distance facilitates knowledge spillovers, absorptive capacity, organizational learning, and resource complementarity leading to faster innovation and exploration successes (Cantner and Meder, 2007; Boschma, 2017). Absorptive capacity is spawned during the learning process in R&D activities (Cohen and Levinthal, 1990) through which firms can recognize new knowledge, assimilate it, and apply it to commercial ends (Gilsing et al., 2008). Existing evidence has shown that the process of knowledge transmission is easier and favorable to industrial developments and regional specialization when technologies are similar (Caviggioli et al., 2023). In contrast, unrelated technologies consist of combinations of unfamiliar knowledge fields making its diversification strategy riskier and more costly. Hence, some scholars even seek answers regarding how unrelated variety increases the occurrence of technological breakthroughs (Saviotti and Frenken, 2008; Castaldi et al., 2015). Nonetheless, most breakthrough patents are not purely unrelated technologies but mixed with the related (existing) ones (Boschma et al., 2023).

Therefore, the degree of technological distance depends on the weighted combination of related and unrelated technologies. Arguably, short technological distance may increase knowledge homogeneity and reduce the value of innovation (Guan and Yan, 2016). However, in the context of green innovation and AI technologies, enterprises bear higher market failure risks, R&D and opportunity costs, and slower returns when integrating complex AI technologies (Ning and Guo, 2022) due to stringent regulatory requirements, stakeholder expectations, and customer demands. Therefore, many enterprises may only attempt to reach the bare minimum (legitimacy) requirement by pursuing easier approaches, i.e., integrating most-related AI technologies, to boost green innovation performance and green reputations.

**Hypothesis 1:** Green-AI technological distance negatively impacts enterprises' green innovation performance.

### 2.3 The moderating role of green technological specialization

The relationship between green-AI technological distance and innovation performance may not be uniform across all enterprises. While many firms tend to pursue the easier path of adopting related technologies, some enterprises are capable of recombining complex technologies (Neffke et al., 2018) and reaping higher benefits (Boschma et al., 2023). This section questions what kind of enterprises are likely to opt for distant AI technologies.

Green technological specialization plays a crucial moderating role in this relationship. SGTEs are motivated to integrate proximate AI technologies due to two key factors: similar collaborative agents and risk perception. Rooted in a resource-based view, SGTEs benefit from entering related digital technologies as they attract specific and similar collaborative agents (Bathelt et al., 2012, p. 72–76; Colombelli et al., 2014) to increase economies of scale, lowering infrastructure and labor costs (Malmberg and Maskell, 1997). As specialization connotes the specificity of resources shared, the chance to cooperate increases when there is a certain technological overlap between the potential partners. At the regional level, specialized areas tend to have high competitive pressure due to continuous knowledge upgrading, thus requiring multiple dimensions of proximity to establish trust relationships (Sánchez-García et al., 2023). Thus, SGTEs with advanced technological sophistication may face difficulties in matching distant digital technologies with less complementary infrastructure and resources.

Risk perception correlates with high technological complexity or distance. In SGTEs, certain technologies are highly invested in and thereby restrict the organization's cognition on technological exploration and exploitation. In green digital innovation, organizations that perceive the outcome to be unpredictable and risky tend to solve problems less effectively (Yin and Yu, 2022), and the success rate of green technology innovation is negatively correlated with managers' loss aversion (Li et al., 2023). High specialization implies that organizations pay more attention or share more responsibility in addressing certain environmental problems and social pressures and adhering to environmental regulations which reduce their tolerance to risks. It is then argued that SGTEs are more risk averse as they are not able to spread the R&D costs and probability of successful outcomes to varied (unspecialized) technology fields.

Therefore, this section argues that SGTEs benefit significantly from seeking easily deployable AI technology in the green sector, driven by the attraction of similar collaborative agents and risk perception.

**Hypothesis 2:** Specialization in green technologies negatively moderates the relationship between green-AI technological distance and enterprises' innovation performance.

## 3 Materials and methods

### 3.1 Sample selection and data sources

To retrieve nationwide information on Chinese firms' patents and financial information, three Bureau van Dijk (BvD) sources are used: Orbis (firm financial information), Orbis Intellectual Property (IP) (patent information), and Orbis-Zephyr (merger and acquisition). The databases cover patents filed in eight key jurisdictions and merger and acquisition events, including public and private sectors in China. The dataset covers the period from 2011 to 2021, following the 12th Five-Year Plan (FYP), and is based on the first filing (priority) date. It includes both granted and non-granted patents while excluding duplicated patents filed in different patent offices by utilizing a patent family identifier.

To account for the presence of subsidiaries within a corporation, which may have different focuses such as marketing, R&D, or other divisions, a network of firms under the same parent company, known as Global Ultimate Owners (GUOs), is constructed to calculate the total number of patents produced. This study adopts Leusin's (2022) method to construct the datasets. Firms are considered subsidiaries of GUOs if the minimum ownership threshold is 50.01%. The implementation process involves extracting information about the GUOs and subsidiaries from BvD Orbis IP, enabling the construction of an ownership network. Information on patent owners acquired or merged by a GUO or a subsidiary can be found in BvD Orbis Zephyr and merged with the initial dataset. This can be merged with the dataset in the first step. Individual patent owners are removed, retaining only companies. Furthermore, firms within the same corporation, referred to as enterprise *c* hereafter, are assigned a unique ID to facilitate aggregating information such as patents, financial values, and numbers of employees within a single network. These firms are geographically distributed in various cities, with some potentially located outside China but belonging to a Chinese parent company.

Green patents are identified based on the first 4-digit IPC class officially classified by the World Intellectual Property Organization (WIPO).^1^ The green technology sectors can be classified into 38 sub-topics under 7 broad topics. AI patents are filtered by examining whether the patent title, abstracts, claims, and description contain a list of AI-relevant keywords, using AI-specific key terms provided by Leusin et al. (2020).

The resulting panel dataset comprises 435 Chinese enterprises, *c*, that have filed at least one green patent, with the same enterprise potentially producing AI patents or not. Among these enterprises, a total of 83,099 green patents are recorded, with only 77 enterprises filing a total of 4,951 AI patents over the ten-year observation period.^2^ The dataset is cleaned by excluding a single firm that does not form a network, applying forward and backward filling methods to address missing values commonly found in firms' financial datasets, centralizing all variables, and normalizing the specialization index using a logarithmic function to adjust for skewness and reduce heteroskedasticity. However, missing values may still exist due to companies not producing patents in certain years, resulting in an unbalanced panel. The total number of observations in the dataset is 8,928.

### 3.2 Regression model

Hausman's (1978) test suggested that the fixed-effect model is more appropriate than the random-effect model. A two-way fixed effect is applied to remove any variations explained by the enterprise and time. The model is specified as follows:


$$\begin{array}{l} Total\;number\;of\;green\;patents_{c,t}=Technological\;distance_{i,t}\\ \times Green\;specialization_{c,t}+Network\;size_{c,t}+Employee\;size_{c,t}\\ +\;Turnover_{c,t}+Total\;patent\;number_{c,t}+Total\;assets_{c,t}+\varphi_{c}\\ +\;\alpha_{t}+\varepsilon_{c,t} \end{array}$$

Where *c* is the enterprise, *i* is the technology sector, and *t* is time. φ_*c*_ is the firm-fixed effect. α_*t*_ is the time-fixed effect. ε_*c, t*_ is the error term of the regression model. This model originally faces a reliability issue with negative R^2^ because the technological distance_i, t_ does not vary across firms, unlisske other variables. Nevertheless, running a fixed effect 2SLS model with instrumental variables eliminates this issue.

### 3.3 Variables

#### 3.3.1 Dependent variable

An enterprise's green patent output (*Green Patents*) is used as a proxy of green innovation performance. In the smart specialization and green innovation literature, the number of green patents is commonly used as a determinant of innovation performance and is a robust measurement in the context of GPTs and high-tech industries (Hagedoorn and Cloodt, 2003; He and Su, 2022; Iacobucci and Perugini, 2023).

#### 3.3.2 Independent variable

Green-AI technological distance (*Technological Distance*) is expressed as the difference between the relatedness density of green and AI technologies in the Smart Specialization framework. Technological relatedness refers to the extent to which patents are included in the same technology classifications, which has been a common method (Guan and Yan, 2016; Li and Rigby, 2023). While such literature explores technological relatedness among all general technologies or in certain industries, none has computed technological distance between two separate technological domains, i.e., green and AI technological domains. Green technology has a total of 38 sub-topics (AI is the 39th sub-topic; *i* = *39*) where 435 enterprises may be involved or not involved in the innovation at all and simultaneously participate in a variety of topics. Thus, some pairs of sub-topics are highly related while some are not.

Relatedness density (RD) is first calculated for each enterprise in each green technology sub-topic. To calculate the average technological distance between green and AI technologies with differential degrees of overlapping technology classifications across every sub-topic, computing the Mean Average Error (MAE) between the relatedness density of green IPC sectors and the AI sector serves as a proxy for their technological distance (Leusin, 2022). Relatedness density (RD) is the technological relatedness of technology *i* to all other technology *j* in which enterprise *c* exhibits revealed technological advantage (RTA) (*x*_*i, c, t*_). *EconGeo* package for patent database is used to compute RD (Balland, 2017):


$$\begin{array}{l} RD_{i,c,t}=\frac{\underset{j\;\in c,\;j\;\neq i}{\sum }x_{i,c,t}\;*\;\varphi_{i,j,t}}{\underset{j\;\neq i}{\sum }\;\varphi_{i,j,t}}\times 100 \end{array}$$

Since RD and the relatedness score (φ_*i, j, t*_) calculate the proximity (reverse of technological distance) between technology *i* to all other technologies, Leusin (2022) adopted MAE to specifically measure the difference between the relatedness density of a given green technology sector *i* and the AI cluster, divided by the total number of enterprises (*N*). The formula is as follows:


$$\begin{array}{l} MAE_{i,t}=\;\frac{1}{N}\overset{n}{\underset{j=1}{\sum }}|RD_{green,j}-RD_{AI,j}| \end{array}$$

#### 3.3.3 Moderating variable

*Green Specialization* is measured by the *coefficient of specialization*, also known as a *location quotient*. It measures the degree of a firm's specialization in technology *i* relative to the standard and the degree of concentration of the industry (Gomez and Stair, 2017). Differing from other methods that measure concentration within a specific industry such as the Herfindahl index, the specialization index measures an enterprise's distribution of technologies enterprises by industrial sectors (technological sub-topics) relative to the reference enterprises. The coefficient has an application in identifying key industries that contribute to the national economy rather than the competition strategy as provided by the Herfindahl index. The maximum value corresponds to a situation when a firm devotes its business entirely to an industry that is not present in any other enterprise at time *t*. The *EconGeo* package is used to compute the specialization score (Balland, 2017):


$$\begin{array}{l} Green\;Specialization_{c,t}=Average\left(\frac{\frac{patents_{c,i,t}}{\underset{i}{\sum }patents_{c,t}}}{\frac{\underset{c}{\sum }patents_{c,i,t}}{\underset{c}{\sum }\underset{i}{\sum }patents_{t}}}\right) \end{array}$$

Where the numerator is the enterprise's share of total patents in technology *i*. The denominator is the share of total patents in enterprise *c* in the whole economy (overall enterprises). If the numerator is larger than the denominator, the specialization value is bigger which indicates the enterprise's high level of specialization in certain technology.

#### 3.3.4 Control variables

Control variables include enterprises' general and financial characteristics, as well as other innovation indicators. The general characteristics encompass the total number of companies (*Network size*) and the total number of employees (*Employee size*) of enterprise *c*. The size not only controls the differences in firms' capabilities to produce patents but also the legitimacy pressures firms face, i.e., larger firms tend to receive high attention from the government and media coverage (Li et al., 2018) and the entry and technological performance (Leten et al., 2016).

Financial and innovation variables include their total income (*Turnover*) and *Total Assets*. Other innovation-related factors considered are the combined green and non-green patents (*Total Patent Numbers*) and the total R&D expenditure (*R&D Intensity*). Higher turnover implies that companies have more profits to be pooled into the R&D investment which, therefore, must be controlled for (Park et al., 2018). Assets can be redeployed in firms' path-branching process where firms build on their capabilities in existing industries (Grillitsch et al., 2018). Patents are the products of R&D expenditure, and other non-green inventions may also affect enterprises' productivity to invent green patents.

## 4 Results

The study reports 3 main results in Section 4. The descriptive evidence in section 4.1 illustrates the heterogeneity of green sectors and enterprises' specialization patterns. Section 4.2 introduces regression analysis to examine the role of SGTE across a spectrum of green-AI technological distance. Section 4.3 reports additional empirical tests.

### 4.1 Descriptive evidence

**Figure 1:**
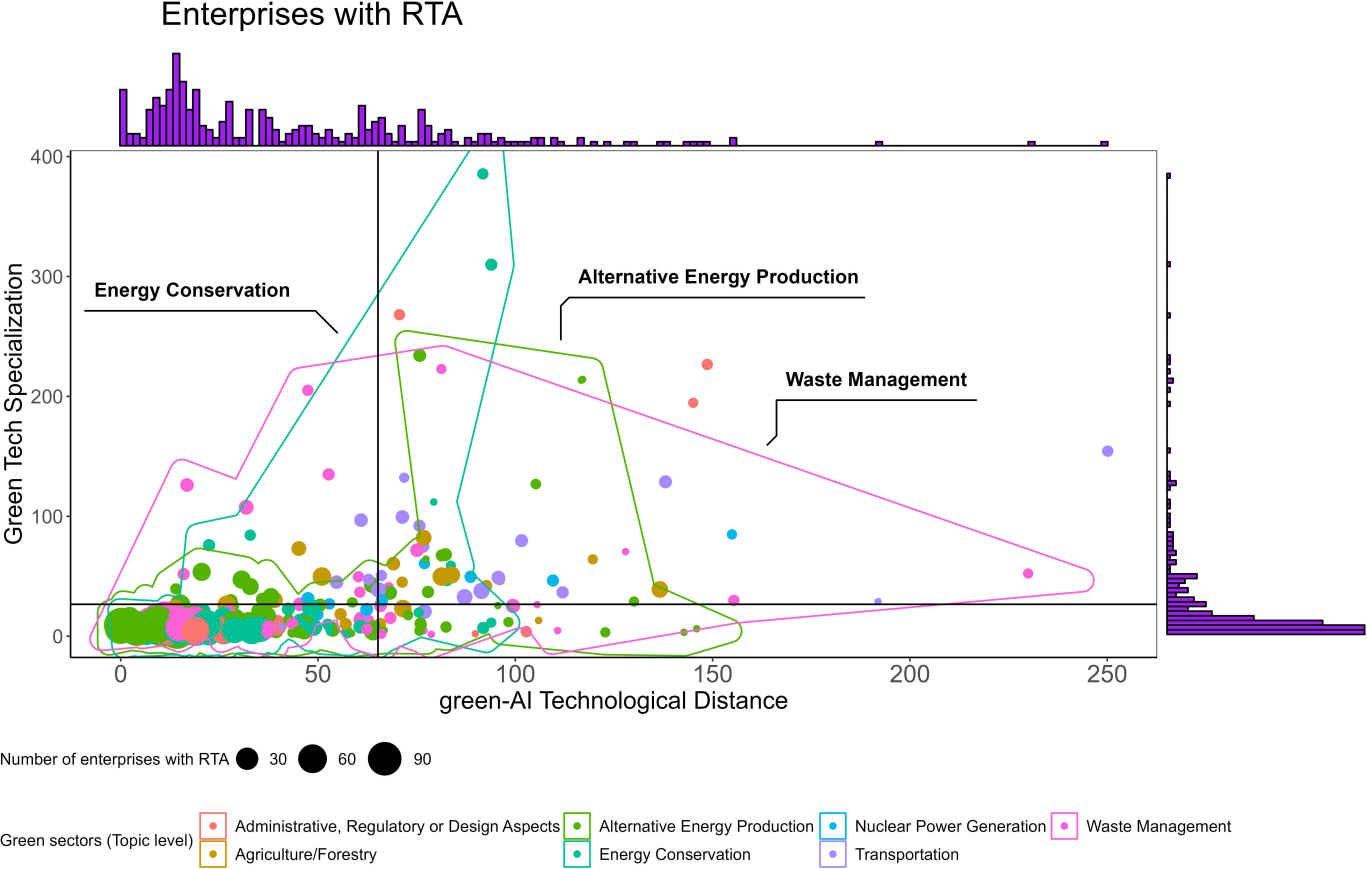
The number of enterprises with RTA and the relationship between green technological specialization and green-AI technological distance (2011–2020). The figure shows that three green sectors “Energy conservation,” “Alternative energy production,” and “Waste management” capture RTA in green technologies that are distant from AI at a high specialization degree.

**Figure 2:**
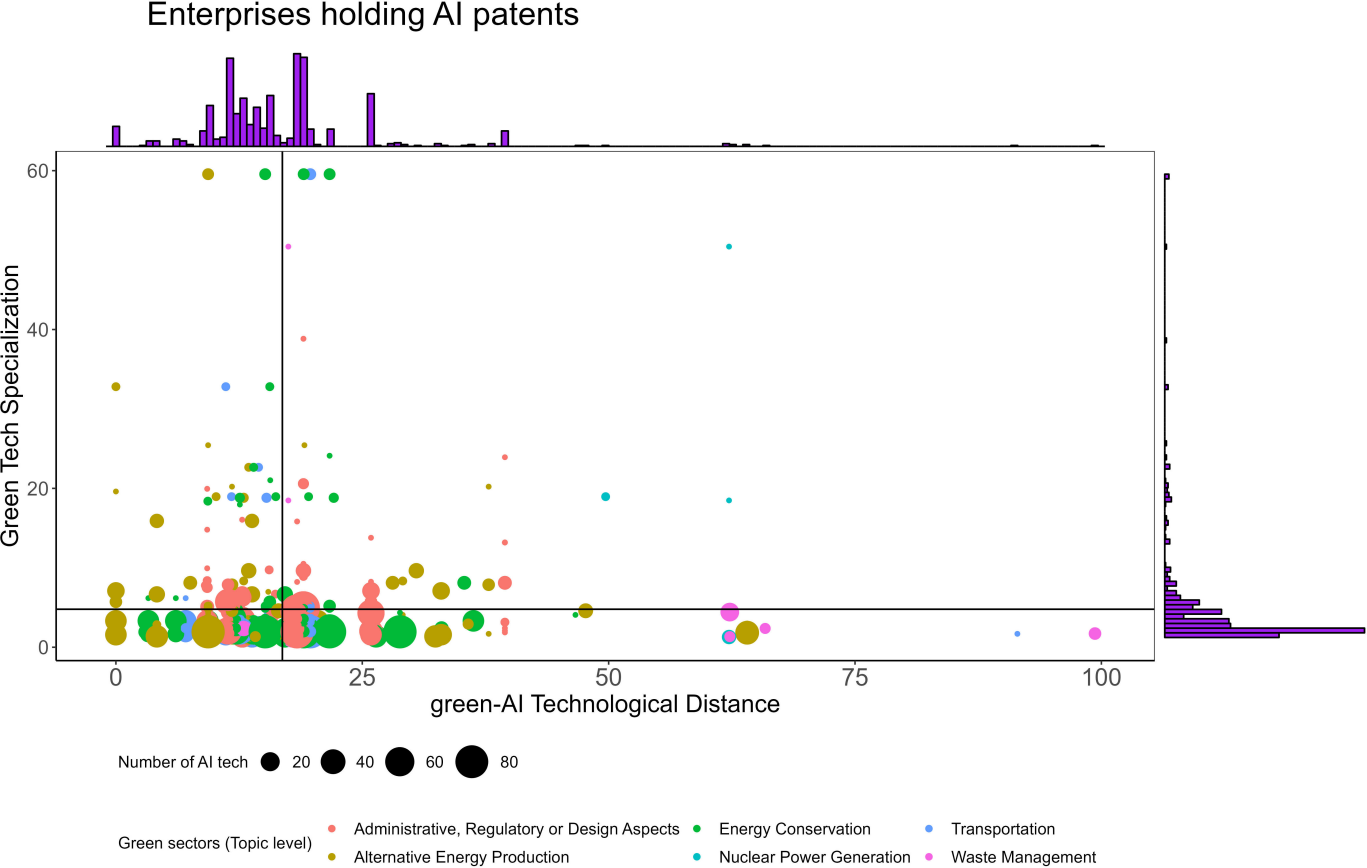
The number of AI technologies and the relationship between green technological specialization and green-AI technological distance (2011–2020). The figure shows that enterprises with high numbers of AI patents tend to be less specialized (diversify more).

Figure 1 illustrates four quadrants that depict technologically specialized (SGTE) and non-specialized enterprises with revealed technological advantage (RTA) in the green industry, based on the green-AI technological distance. Each quadrant highlights the trade-off between gaining specialization and integrating proximate AI technologies. Figure 2 provides a visual representation of the number of AI patents created by enterprises across all four quadrants.

The findings from Figure 1 suggest that capturing green RTA is relatively easier in all industries within the lower-left quadrant, where firms have low specialization and a short green-AI distance. Additionally, several sectors are situated in the upper-right quadrant, such as “Energy conservation,” “Alternative Energy Production,” “Waste Management,” and “Transportation.” These sectors have successfully captured RTA in green technologies that are distant from AI. Notably, the first three sectors span across all four quadrants, indicating a diverse range of candidates within those sectors. On the other hand, the lower-right quadrant consists of sectors with low specialization and a long green-AI distance. Enterprises in this segment tend to gain less RTA and demonstrate lower innovation performance, as further analyzed in the subsequent section.

Figure 2 highlights that enterprises with a high number of AI patents predominantly occupy the lower two quadrants, indicating a lower specialization in the green domain. In contrast, SGTEs in the upper quadrants tend to have fewer AI inventions, which may explain their inclination toward acquiring RTA in distant and complex AI technologies, as depicted in Figure 1.

It is important to note that the “Agriculture/Forestry” sector is absent from Figure 2 due to the exclusion of a single firm from the dataset. Statistical evidence reveals that over 40% of companies in China have not prioritized AI as a strategic focus (Barton et al., 2017). Similarly, the “Nuclear Power Generation” sector has minimal representation in AI innovation within China. This can be attributed to the sector's high localization and regulation by the State Council (Andrews-Speed, 2023). Typically, R&D development in this sector involves collaborations with Chinese research institutes and universities, aiming to stimulate product exportation in the nuclear power domain.

### 4.2 Regression analysis results

Table 1 presents the descriptive statistics of the variables and the correlation matrix for the variables. All variables are centralized with a mean of zero. The VIF is less than the threshold value of 10, suggesting no serious multicollinearity issues. Correlation between variables is highly significant. For example, enterprises with high *R&D Intensity, Network size, Employee size, Turnover*, and *Assets* tend to generate high green innovation performance *(Green Patents)*. Within this, *Network Size, Employee Size*, and *Turnover* are associated with increased technological distance. Meanwhile, *Green Specialization* negatively correlates with *Green Patents* and *Network size and Employee size*, suggesting that enterprises that diversify their green portfolios and target less monopolistic green sectors tend to be large enterprises that could generate high numbers of green patents. Small enterprises thus have a high tendency to be SGTEs which are typically involved in highly specialized green activities.

**Table 1:** Descriptive statistics and pairwise correlation matrix.

**Variable**	**Mean**	**SD**	**Min**	**Max**	**VIF**	**1**	**2**	**3**	**4**	**5**	**6**	**7**	**8**
Green patents	0.00	89.31	−49.81	746.19									
Technological distance	0.00	18.59	−18.52	231.53	1.453	0.036^***^							
Green specialization	0.00	0.99	−1.34	5.50	1.220	−0.24^***^	0.24^***^						
Network size	0.00	5.83	−3.53	45.47	2.254	0.61^***^	0.034^***^	−0.22^***^					
Employee size	0.00	4.44	−2.32	33.23	1.615	0.4^***^	0.044^***^	−0.15^***^	0.61^***^				
Turnover	0.00	107.66	−39.35	832.93	1.943	0.36^***^	0.017^*^	−0.17^***^	0.64^***^	0.43^***^			
Total patent number	0.00	37.89	−3.66	1329.52	1.007	0.36^***^	−0.0013	−0.053^***^	0.045^***^	0.07^***^	0.025^**^		
Assets	0.00	367.01	−75.44	6846.64	1.176	0.1^***^	−0.00007	−0.092^***^	0.27^***^	0.18^***^	0.38^***^	0.0025	
R&D intensity	0.00	18.82	−8.07	185.97	1.110	0.091^***^	0.012	−0.15^***^	0.2^***^	0.089^***^	0.29^***^	0.00019	0.12^***^

^*^p < 0.1; ^**^p < 0.05; ^***^p < 0.01. All variables are mean–centered.

Table 2 provides a summary of the time-series regression analysis conducted to test the first and second hypotheses. The regression compares the 2SLS method with the ordinary least squares (OLS) method to address the potential endogeneity problem arising from the influence of enterprise characteristics on the explanatory and moderating variables. Durbin and Wu-Hausman test was performed to test the consistency of both models. In Table 2, the explanatory variables are regressed on their instruments, which include the inverse of *Technological Distance* and 2-year lagged *Green Specialization* for the moderator. All instruments solely affect the outcome through the independent variables and are not weak (*p* < 0.05), satisfying instruments' conditions in 2SLS. The Durbin Wu-Hausman test fails to reject the null hypothesis of inconsistency between the two models (*p* > 0.1), indicating that the OLS estimates do not significantly differ from the 2SLS estimates and thus can be used to interpret the results. Furthermore, the OLS regressions in both Models 1 and 2 exhibit poor fit with the data, as indicated by negative adjusted R-squared values, which are resolved when conducting the same regression with the 2SLS method.

**Table 2:** Regression results for direct and moderation effects.

	**Green innovation performance**			
	**Model 1**		**Model 2**	
	**OLS**	**2SLS**	**OLS**	**2SLS**
Technological distance	−0.151^**^	−0.369^***^	−0.223^**^	−0.395^***^
	(0.070)	(0.131)	(0.103)	(0.153)
Green specialization			−3.072^***^	−37.060^**^
			(0.856)	(16.480)
Network size	5.482^***^	8.575^***^	5.410^***^	7.331^***^
	(0.412)	(0.415)	(0.411)	(0.668)
Employee size	0.783^***^	0.546^**^	0.770^***^	0.297
	(0.184)	(0.260)	(0.183)	(0.295)
Turnover	−0.068^***^	−0.032^**^	−0.066^***^	−0.055^***^
	(0.020)	(0.015)	(0.020)	(0.021)
Total patent number	0.493^***^	0.584^***^	0.492^***^	0.567^***^
	(0.025)	(0.056)	(0.025)	(0.053)
Total assets	0.002^**^	−0.007^***^	0.002^**^	−0.007^***^
	(0.001)	(0.001)	(0.001)	(0.001)
Total R&D intensity	0.038	−0.005	0.042	−0.103
	(0.034)	(0.059)	(0.034)	(0.078)
Technological distance × green specialization			0.059^**^	0.270^*^
			(0.030)	(0.138)
Weak instruments		0.000^***^		0.000^***^
Wu–Hausman		0.254		0.173
Observations	8,928	8,168	8,928	8,166
R^2^	0.246	0.407	0.247	0.341
Adjusted R^2^	−0.133	0.106	−0.131	0.006

^*^p < 0.1; ^**^p < 0.05; ^***^p < 0.01; Robust standard error is reported.

The negative coefficient of green-AI *Technological Distance* is most significant in the 2SLS model (*p* < 0.01), supporting hypothesis 1. The increased significant results suggest that 2SLS effectively eliminates the bias and heterogeneity issues within the sample population (Card, 2001). This pattern applies to Model 2 as well. The reduction of bias and heterogeneity issues is due to the shift of the population group toward the sub-group population with higher-than-average innovation performance when using 2SLS's instruments.^3^ Overall, the first hypothesis is supported, indicating that enterprises generally benefit more, in terms of innovation advantages, from integrating AI technologies that are closer to existing green technologies.

**Figure 3:**
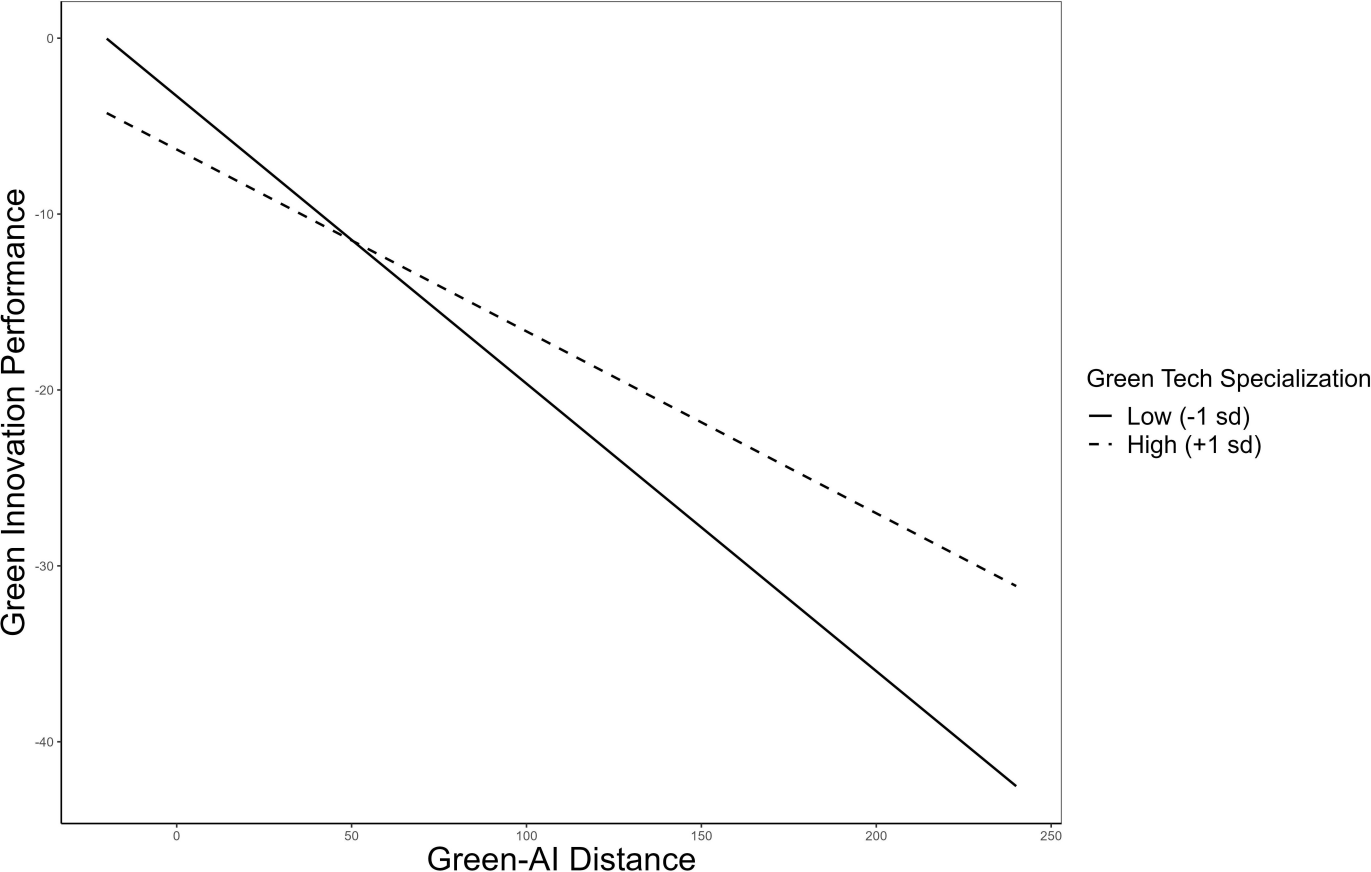
The moderating effect of green technological specialization on the relationship between green-AI technological distance and green innovation performance.

The interaction term of green-AI *Technological Distance* and SGTE significantly relates to green innovation performance. Interestingly, the relationship is contrary to the hypothetical expectation, leading to the rejection of Hypothesis 2. The moderation effect is moderately strong, with *p* < 0.05 (OLS model) and 0.1 (2SLS model) in Model 2. Figure 3 illustrates the interaction results, demonstrating that SGTEs perform better at longer green-AI *Technological Distance*. Therefore, the theoretical assumption regarding their short cognitive proximity and high-risk perception is not supported in the case of Chinese enterprises.

The rejection of the second hypothesis can be attributed to several factors. Firstly, in the digital industry, where technologies are accessible to various types of enterprises, specialized firms have a lower probability of survival (Mangani and Tarrini, 2017), because they are less agile compared to diversified industries when competing for related AI technologies (Fernández et al., 2016). However, under high technological uncertainty, firms may increase their specialization to compensate for the lack of speed, lead-time advantage, or litigation capability (Toh and Kim, 2013). Technological uncertainty may arise, for example, when environmental regulations prompt the government to fund valuable but faltering green projects (Guo et al., 2018), increase accessibility to technical support and subsidies (Zhang and Zhang, 2023), and enhance green legitimacy (Li et al., 2023). These factors act as a risk-mediating buffer, encouraging SGTEs to invest in a highly complex innovation. From a management perspective, a high-risk attitude may not limit managers' open attitude or lower their tolerance for failure in SGTEs. Instead, it might allow them to be more cautious and objective when selecting distant AI technologies (Yin and Yu, 2022).

### 4.3 Robustness checks

To account for the potential influence of variable indicators on the research results, particularly within “green” enterprises, a focus is placed on enterprises from renewable energy sectors within the initial sample data. This selection is made to understand the potential impact on the research findings. As a result, the unbalanced panel data is reduced to 690 observations and 100 enterprises.

The findings presented in Table 3 indicate that the impact of green-AI *Technological Distance* on green innovation performance remains stable and consistent. However, the moderating effect of *Green Specialization* on green innovation performance is not found to be significant. This lack of significance can be attributed to the fact that enterprises from the renewable energy sector exhibit high specialization in specific sub-sectors within the Alternative Energy Production sector, as classified by WIPO's Green IPC category. By reducing the heterogeneity of green sectors through this specialization, the observed effect becomes non-significant.

**Table 3:** Regression results of robustness tests.

**Variables**	**Model 1**	**Model 2**
Technological distance	−0.136^***^	−0.111^**^
	(0.044)	(0.044)
Green specialization		−8.995^***^
		(2.164)
Network size	4.896^***^	4.495^***^
	(0.699)	(0.650)
Employee size	1.159^***^	1.064^***^
	(0.426)	(0.407)
Turnover	−0.139^**^	−0.121^**^
	(0.064)	(0.061)
Total patent number	5.384^***^	5.262^***^
	(1.042)	(1.024)
Total assets	−0.037	−0.041
	(0.050)	(0.048)
Total R&D intensity	0.110	0.108
	(0.169)	(0.163)
Technological distance × green specialization		0.004
		(0.017)
Observations	690	690
R^2^	0.519	0.534
Adjusted R^2^	0.123	0.147

^**^p < 0.05; ^***^p < 0.01; Robust standard error is reported.

### 4.4 Heterogeneity tests

The correlation matrix (Table 1) suggests that enterprises with large networks and employees tend to diversify more complex green patents into a variety of green sectors, reducing their specialization score and increasing the number of green patents produced. Small network/employee enterprises, on the other hand, seem to be highly specialized. The interaction term of regression analysis suggests that specialized enterprises tend to innovate more at a high technological distance, given the network/employee size is constant. To examine which size of enterprises is more significant to the relationship between technological distance, green specialization, and green innovation performance, heterogeneity analyses of enterprises' *Network Size* and *Employee Size* are conducted in Tables 4, 5.

**Table 4:** Heterogeneity tests of enterprises' network size.

	**Green innovation performance**					
	**Large network size**			**Small network size**		
	**Model 1**	**Model 2**	**Model 3**	**Model 4**	**Model 5**	**Model 6**
Technological distance	0.018	0.023	0.023	0.046	0.086^*^	0.174^**^
	(0.093)	(0.051)	(0.094)	(0.051)	(0.051)	(0.083)
Green specialization		−1.059	−1.006		−8.861^***^	−8.403^***^
		(1.084)	(1.084)		(1.084)	(1.120)
Network size	4.349^***^	4.338^***^	4.337^***^	11.350^***^	9.677^***^	9.645^***^
	(0.233)	(1.059)	(0.233)	(1.176)	(1.059)	(1.062)
Employee size	−0.175	−0.186	−0.186	0.784^**^	0.719^*^	0.725^*^
	(0.179)	(0.373)	(0.181)	(0.379)	(0.373)	(0.373)
Turnover	−0.014	−0.014	−0.014	0.034	0.028	0.027
	(0.009)	(0.028)	(0.009)	(0.028)	(0.028)	(0.028)
Total patent number	14.870^***^	14.870^***^	14.870^***^	0.516^***^	0.511^***^	0.511^***^
	(0.536)	(0.033)	(0.536)	(0.035)	(0.033)	(0.034)
Total assets	0.001	0.001	0.001	−0.048^***^	−0.046^***^	−0.045^***^
	(0.001)	(0.015)	(0.001)	(0.015)	(0.015)	(0.015)
Total R&D intensity	−0.448^***^	−0.454^***^	−0.453^***^	0.192^***^	0.178^**^	0.181^***^
	(0.076)	(0.070)	(0.077)	(0.073)	(0.070)	(0.070)
Technological distance × green specialization			−0.013			−0.064^*^
			(0.045)			(0.033)
Observations	2,628	2,628	2,628	6,300	6,300	6,300
R^2^	0.839	0.839	0.839	0.291	0.313	0.315
Adjusted R^2^	0.754	0.754	0.754	−0.054	−0.020	−0.019

^*^p < 0.1; ^**^p < 0.05; ^***^p < 0.01; Robust standard error is reported.

**Table 5:** Heterogeneity tests of enterprises' employee size.

	**Green innovation performance**					
	**Large employee size**			**Small employee size**		
	**Model 1**	**Model 2**	**Model 3**	**Model 4**	**Model 5**	**Model 6**
Technological distance	−0.387^***^	−0.312^**^	−0.401^***^	−0.016	−0.004	0.011
	(0.143)	(0.140)	(0.154)	(0.047)	(0.047)	(0.061)
Green specialization		−11.610^***^	−12.950^***^		−2.732^***^	−2.658^***^
		(2.164)	(2.312)		(0.505)	(0.506)
Network size	8.581^***^	8.193^***^	8.223^***^	6.327^***^	6.217^***^	6.214^***^
	(0.485)	(0.504)	(0.501)	(0.473)	(0.471)	(0.472)
Employee size	1.734^***^	1.756^***^	1.775^***^	−2.215^*^	−2.363^*^	−2.350^*^
	(0.498)	(0.495)	(0.496)	(1.290)	(1.300)	(1.300)
Turnover	−0.064^***^	−0.060^***^	−0.060^***^	0.028^**^	0.023^**^	0.023^**^
	(0.021)	(0.020)	(0.020)	(0.012)	(0.012)	(0.012)
Total Patent number	0.542^***^	0.537^***^	0.537^***^	9.285^***^	9.227^***^	9.226^***^
	(0.044)	(0.043)	(0.043)	(0.894)	(0.893)	(0.893)
Total assets	−0.012^***^	−0.014^***^	−0.014^***^	−0.002^***^	−0.002^**^	−0.002^**^
	(0.002)	(0.002)	(0.002)	(0.001)	(0.001)	(0.001)
Total R&D intensity	−0.637^***^	−0.680^***^	−0.690^***^	−0.062^*^	−0.069^*^	−0.069^*^
	(0.089)	(0.098)	(0.098)	(0.037)	(0.037)	(0.036)
Technological distance × green specialization			0.115^**^			−0.013
			(0.050)			(0.021)
Observations	2,465	2,465	2,465	6,463	6,463	6,463
R^2^	0.438	0.445	0.446	0.659	0.661	0.661
Adjusted R^2^	0.151	0.162	0.163	0.491	0.494	0.494

^*^p < 0.1; ^**^p < 0.05; ^***^p < 0.01; Robust standard error is reported.

#### 4.4.1 Heterogeneity analysis of enterprises' network size

In Table 4, the network size is split into large and small network enterprises, using mean value as a threshold. The impacts of technological distance and its interaction with the moderator are significant only in enterprises with small network sizes. However, the technological distance and green specialization have a positive and negative coefficient at *p* < 0.001 and *p* < 0.1 respectively (Model 6), suggesting that enterprises with fewer subsidiaries or acquired/merged companies tend to produce more green patents when integrating distant AI technologies, and this effect would be reduced if they become more specialized.

#### 4.4.2 Heterogeneity analysis of enterprises' employee size

Similarly, the employee size is split into two groups based on the mean value in Table 5. Contrary to the heterogeneity analysis of network size, the employee size shows a result consistent with the main results in the population of enterprises with large (higher than average) employee sizes (Table 5). Therefore, even if the number of observations in enterprises with large employee sizes is small, the model remains robust in this group. In combination with the previous heterogeneity analysis, the results suggest that diversifying resources to too many companies within the corporation could reduce the R&D efforts in integrating distant AI technologies while increasing the number of employees will benefit the innovation process.

## 5 Discussion

### 5.1 Main findings

The growing concern over climate change has prompted enterprises to incorporate AI technology into the realm of green innovation. However, our understanding of organizational innovation strategies in the green industry is limited. In particular, this research contributes to the green-digital literature, suggesting the appropriate strategies to recombine green and AI technologies for SGTEs and non-specialized enterprises. It also adds evidence to the relatedness and specialization theories, unveiling the pros and cons of becoming specialized in green technologies.

The first finding supports the existing relatedness theory where short-distant technologies facilitate faster innovation performance. These are enterprises that aim to capture a larger share of RTA. The second finding rejects the initial hypothesis, showing that SGTEs produce more green patents when integrating distant AI technologies. This finding leads to two theoretical explanations. First, SGTEs face challenges in commercializing technologies swiftly (Toh and Kim, 2013; Mangani and Tarrini, 2017), so they compensate by taking advantage of special circumstances, such as environmental regulations and government incentives, to integrate distant AI technologies that are deemed to contribute more to the country's innovation. These advantages include eligibility to obtain government R&D funding or access to technical support and enhanced green legitimacy (Li et al., 2023). Particularly, a recent study reports that government subsidies in China tend to promote enterprises with specialization in a specific sector of green technologies but not those with mixed focuses (Zhang and Zhang, 2023). The subsidies act as a risk-mediating buffer, encouraging SGTEs to invest in a highly complex innovation. Second, SGTE's high-risk attitude may increase the cautiousness of decision-making, resulting in fewer errors and superior innovation performance when integrating distant AI technologies (Yin and Yu, 2022).

In the descriptive evidence, it is observed that enterprises generate a higher number of AI patents when they exhibit lower specialization (greater diversification) in green technologies. This implies that the use of AI technologies expands the breadth of knowledge items horizontally. However, as firms allocate time and resources across numerous technology classes, their specialization becomes compromised. This poses questions regarding whether specialization should be emphasized more as a long-term strategy for China's green development (Conti et al., 2019).

### 5.2 Limitations and future research

The present study exhibits several theoretical and methodological limitations that should be acknowledged. Theoretical limitations encompass two aspects, while methodological limitations can be categorized into three areas.

The first theoretical limitation concerns the generalizability of the research in the context beyond Chinese firms. Chinese entrepreneurs' technological diversification strategies could be unique due to specific external influences such as political systems, government intervention, and historical background. Known for its leadership in green industries, China serves as a model for other emerging economies to apply (Hain et al., 2021). Therefore, replicating the study in other contexts such as advanced countries or least developing countries would further contribute to the relevant literature.

The second theoretical limitation concerns the direct translation of invention as a sole product of the innovation process. Innovation, as defined, encompasses the entirety of production processes, which may not necessarily be fully captured by patents. It is important to recognize that firms strategically opt for intellectual property (IP) rights to yield greater benefits for their businesses (Lanjouw and Schankerman, 2001). Nonetheless, the advantages of IP rights outweigh the disadvantages, as they foster progress in scientific innovation and establish market foundations (Spulber, 2015). Furthermore, technological advancements do not guarantee a substantial reduction in carbon pollution. Firms engaged in global value chains, particularly those operating in developing countries, often cause more environmental issues (Wang et al., 2020). Therefore, it is recommended that future research assesses the impact of variable indicators on sustainable development or environmental issues.

Regarding methodological limitations, the absence of spatial characteristics in the data samples precludes the inclusion of China's regions as control variables. In other words, this study defines an enterprise as a network of firms from a variety of divisions segregated across different geographical locations. Considering that green development varies across regions and governmental institutions, this limitation prevents the model from achieving greater stability (He and Su, 2022; Yin et al., 2023). Consequently, the study employs 2SLS instruments as discussed in the main analysis to address this issue. Nevertheless, the sample data could be potentially biased toward Chinese enterprises located in the Eastern region, where technologies are well-developed.

Furthermore, despite the elimination of patent owners filed as individuals in the data sample, universities that actively collaborate with companies are included. Additionally, the algorithm used to construct a network of firms sharing the same patents is static rather than dynamic. For instance, the algorithm assigns inventor A as a partner of inventor B since 2011, even though in reality they were separate entities that year but later became sister companies or were acquired by others. This is because the same identifiers must be assigned to enterprises even though they have been mobilized or removed from the networks. Issues are rectified by combining the dataset with the merger and acquisition database which provides more precise information, making the network less static.

### 5.3 Contributions and implications

This study contributes theoretical and practical insights to the literature on relatedness and green digitalization, specifically within the context of developing countries. The findings highlight that SGTEs, which hypothetically have short cognitive proximity, exhibit significant innovation when incorporating complex AI technologies. However, this pattern is not without its limitations. On one hand, only a limited number of SGTEs can effectively integrate AI technologies that are less closely related to their current green technologies. On the other hand, enterprises that encompass a broader range of technology sectors tend to compromise their specialization advantages by pursuing less complex AI technologies, thereby overlooking the transformative potential of distant AI technologies.

The study offers practical recommendations for policymakers and organizational managers or industry practitioners. It emphasizes the need to incentivize non-specialized enterprises to explore and engage with distant AI knowledge domains with support from policymakers, other than incentivizing the ‘pure' green sectors. Providing R&D subsidies to non-specialized enterprises will encourage them to take more risks in integrating complex AI technology, promoting the development of green industries. Meanwhile, recent news reports that EV cars have been over-supplied and caused bankruptcy, affecting the industrial supply chain (Bloomberg News, 2023). With the lack of government support, SGTEs are most susceptible to market change due to their diversification risks. Therefore, SGTEs with high potential should receive appropriate protection. For industry practitioners, the provision of open-collaboration platforms and maintaining competitiveness is recommended. Creating spillover channels from specialized to diversified firms (and vice versa) is a low-risk strategy that elevates the complexity of the nation's knowledge of green technologies. Simultaneously, SGTEs could take a leadership role in searching for AI breakthroughs or collaborative partners outside China. For instance, selecting potential partners with a small overlap of technological knowledge (experience) may reduce the probability of cooperating due to cultural and professional differences and increase technological values once accomplished. Overall, the integration of complex AI technology would contribute more to net-zero transitions since many green industries are still seeking better technologies to solve environmental problems.

## 6 Conclusion

This research addresses the research question of “What is the role of AI in enhancing organizational green innovation performance, and how does it favor an organization's green specialization?”. The research confirms the role of relatedness on innovation performance and reveals that green specialization performs more innovatively when integrating distant AI technologies into green innovation.

The study adds a different insight into the roles of green specialization in AI technological integration. It puts forward several speculations to explain the phenomenon of why SGTEs tend to experience a decline in their innovation advantages when integrating related AI technologies but gain incentives to generate breakthrough innovations by integrating distant AI technologies. As powerful digital technology becomes pervasive, enterprises that diversify their technological focuses tend to gain the greatest advantages from integrating AI close to their knowledge. However, SGTEs possess a specific competitive advantage, perhaps due to high-risk perception and national support, creating incentives for them to pursue breakthrough innovations with distant AI technologies.

Therefore, the ability to enact policies and management practices favorable to SGTEs and non-specialized enterprises' innovative behavior could drive the nation closer to sustainable goals. This requires the coordination of all stakeholders. For example, the government's provision of R&D incentives and collaboration between public and private sectors to create spillovers and incentivize firms to engage more in recent, complex AI technologies. Complex technologies are valuable assets that can be easily innovated by SGTEs. Hence, SGTEs serve as an example of good management practices, addressing complex environmental problems that cannot yet be remedied by general (short technological distance) AI technologies.

Admittedly, the study subjects to a few limitations in terms of generalizability and the use of patent data as a sole determination of enterprises' innovation development. The study also poses some methodological limitations, which call for a replication of research experiments with better methodologies. Future improvement includes exploring the phenomena found in this research in contexts outside China or across Chinese provinces with different institutional qualities and exploring the technological growth's impacts on ecological efficiency.

In light of these findings, it is hoped that this research will stimulate further interest in future studies on the twin transition of green and AI technologies, as well as the mechanisms underlying firms' digital integration in the context of sustainability.

## Data availability statement

The original contributions presented in the study are included in the article/supplementary material, further inquiries can be directed to the corresponding author.

## Author contributions

SK is responsible for conceiving the study, collecting the data, developing the analyses, and writing the manuscript.

## Footnotes

^1^Green IPC category is provided in the following website: https://www.wipo.int/classifications/ipc/green-inventory/home.

^2^The total number of AI-related patents in China is 72,802 which is registered by a total of 5,385 Chinese individual companies.

^3^OLS only estimates the average difference in green innovation performance for each unit difference of green-AI *Technological Distance* across the entire population.
